# Lung-targeted SERCA2a Gene Therapy: From Discovery to Therapeutic Application in Bleomycin-Induced Pulmonary Fibrosis

**Published:** 2020-05-06

**Authors:** Malik Bisserier, Lahouaria Hadri

**Affiliations:** Cardiovascular Research Center, Icahn School of Medicine at Mount Sinai, New York, NY 10029, USA

**Keywords:** SERCA2a, Lung Fibrosis, AAV1, Gene Therapy

## Abstract

Idiopathic pulmonary fibrosis (IPF) is an interstitial lung disease characterized by an accumulation of scar tissue within the lungs and the common presence of usual interstitial pneumonia. Unfortunately, only a few FDA-approved therapeutic options are currently available for the treatment of IPF and IPF remains associated with poor prognosis. Therefore, the identification of new pharmacological targets and strategies are critical for the treatment of IPF. This commentary aims to further discuss the role of sarcoplasmic reticulum Ca^2+^-ATPase 2a and its downstream signaling in IPF. Finally, this commentary offers new insights and perspectives regarding the therapeutic potential of AAV-mediated SERCA2A gene therapy as an emerging therapy for respiratory diseases.

## Commentary

Idiopathic pulmonary fibrosis (IPF) is a chronic, progressive, devastating, and rare lung disease [1]. IPF is characterized by usual interstitial pneumonia and represents the most common idiopathic interstitial lung disease. While IPF primarily affects middle-aged and older adults [2], several risk factors have been identified over the past decades, such as genetic predisposition, cigarette smoking, and chronic viral infections [3]. Despite the identification of new therapeutic targets and the development of several anti-fibrotic drugs, only a few therapeutic options have been FDA-approved and are currently available for the treatment of IPF [4]. Therefore, there is a clear and urgent need for identifying and developing novel effective therapies for IPF disease.

**Figure 1:**
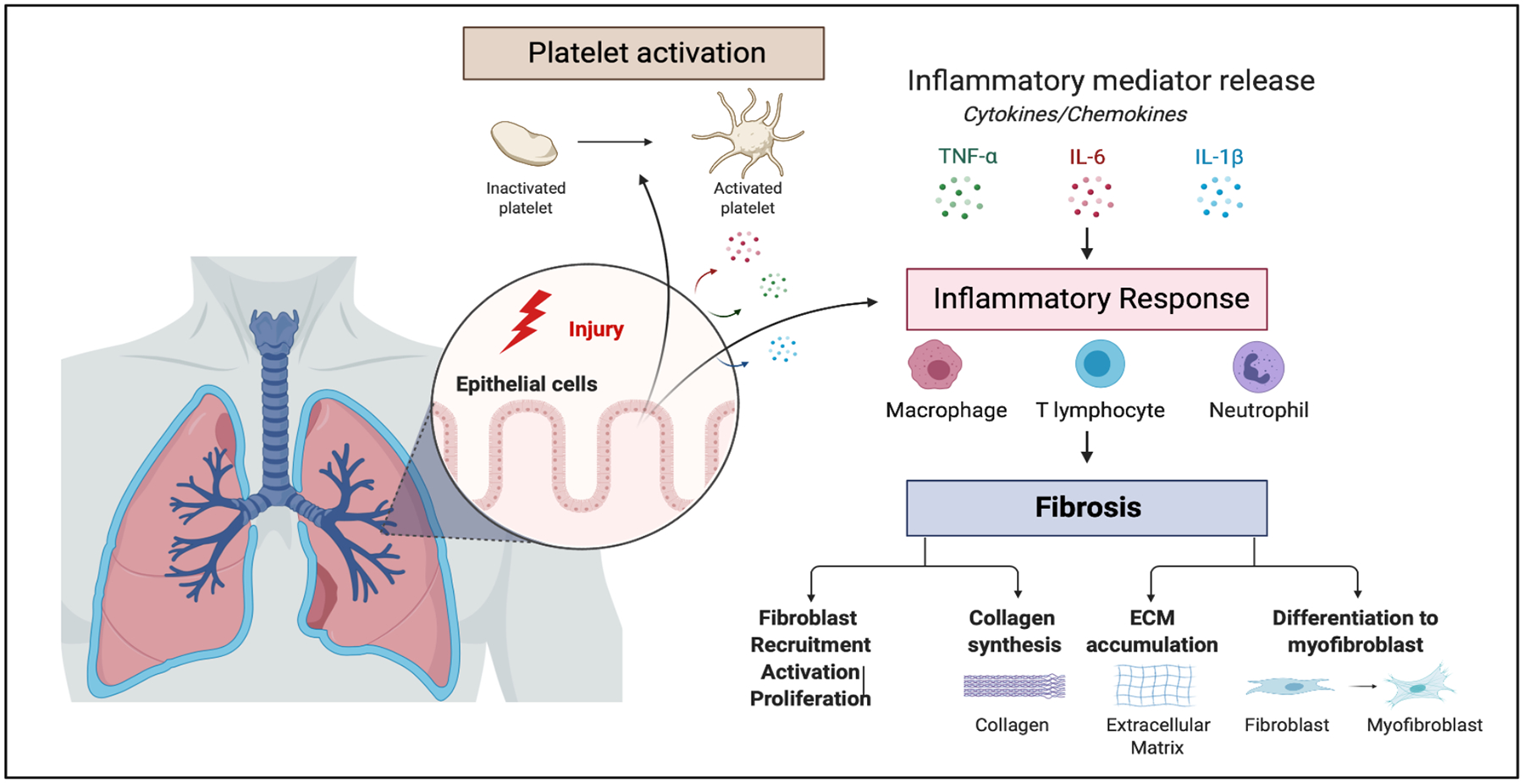
Pathobiology of idiopathic pulmonary fibrosis. Alveolar epithelial micro-injury induces a maladaptive wound healing response, which is characterized by the progressive accumulation of scar tissue. The dysregulated alveolar repair response is associated with the secretion of inflammatory mediators, including cytokines and chemokines, which lead to the platelet activation and mediate the recruitment of inflammatory cells such as macrophages, lymphocytes and neutrophil. These inflammatory cells release pro-fibrotic cytokines that recruit and activate fibroblasts, potentiate collagen synthesis, accumulation of extracellular matrix (ECM) components as well as promoting differentiation of fibroblast into myofibroblasts. These biological processes contribute to the pathological remodeling of lung architecture, which may result in the obliteration of lung tissue and ultimately respiratory failure. Created with BioRender.com.

The pathogenesis of IPF is usually initiated by micro-injuries of the lung alveolar epithelium with abnormal reepithelization and repair mechanisms that stimulate a dysregulated wound healing response [5]. Progressive accumulation of scar tissue is associated with the recruitment and activation of myofibroblasts, activation of fibroproliferative processes, as well as an excessive deposition of extracellular matrix components, and abnormal accumulation of collagen (Figure 1) [6]. These biological processes lead to a pathological remodeling of lung architecture, resulting in the obliteration of lung tissue, respiratory failure, and ultimately death [7]. To date, despite extensive research efforts in experimental and clinical studies, PF remains an increasing cause of morbidity with a median survival from diagnosis of 2.8–4.2 years and an overall 5-year survival approaching 20% [8]. Therefore, offering more effective and reliable therapeutic options for IPF is of the utmost importance.

The BLM-induced pulmonary fibrosis model is known as the most common experimental model of human IPF [9–11]. BLM delivery induces lung injury with a subsequent fibroproliferative response in mice by causing cellular damage to the alveolar and endothelial cells and other cell types, leading to the production of cytokines and profibrotic mediators such as TGF-β and IL-6 [9,11]. It is now well-accepted that the BLM-induced lung injury model is composed of two phases: an inflammatory phase characterized by recruitment of leukocytes within one week; and a second, fibrotic phase characterized by increased fibroblast proliferation and differentiation to myofibroblast, as well as synthesis of ECM during the second week [9,12]. The use of genetically-engineered mice subjected to BLM instillation has provided significant insights toward the identification of new pharmacological targets and the development of novel interventional techniques for treating patients with IPF.

During the initiation of fibrotic processes, activation of inflammatory and epithelial cells increases the production and release of the pro-fibrosis mediator transforming growth factor (TGF-β) [6]. TGF-β, one of the main profibrogenic cytokines, is a prominent regulator involved in the development of fibrosis in different organs [13]. In the lungs, it plays a critical role in the fibroblast differentiation into myofibroblasts, collagen production, and, therefore, loss of lung elasticity and impairment of the respiratory function [13]. Pre-clinical studies have reported beneficial effects of TGF-β inhibition in bleomycin-induced lung fibrosis [14,15]. For example, pharmacological treatment with P17 (a TGF-β inhibitor peptide) in mice significantly inhibits the progression of lung fibrosis by blocking the SMAD signaling [14]. Similarly, developmental endothelial locus-1 (Del-1, an endogenous inhibitor of TGF-β signaling) attenuates fibrosis in mice by inhibiting αv integrin-mediated activation of TGF-β [15].

Early studies have reported the presence of cellular inflammation in the lung parenchyma in patients with lung fibrosis as a consistent feature [16]. Indeed, histological analysis has revealed an accumulation of lymphocytes, macrophages, eosinophils, and neutrophils as well as the presence of lymphoid follicles with germinal centers in IPF patients [17,18]. These early findings suggest that inflammation may play a critical role during the initiation phase in IPF patients. However, as the use of traditional immunosuppressive therapies such as interferon-gamma, anti-tumor necrosis factor-alpha monoclonal antibody, or low dose of Prednisone and Azathioprine, have failed to improve lung function in IPF patients, the role of inflammation in IPF remains unclear [19]. Moreover, the lack of correlation between the level of inflammatory markers and the severity of IPF strengthens the hypothesis that inflammatory processes may be a secondary feature instead of an initiator event in IPF [19]. Increasing evidence suggests that epithelial injury and aberrant wound healing might be the primary defect in IPF [20].

Interleukin-6 (IL-6) is a pleiotropic pro-inflammatory cytokine as well as a profibrotic factor in bleomycin (BLM)-induced lung injuries [21,22]. In 2008, Saito and colleagues investigated the role of IL-6 in the lung inflammatory changes induced by BLM using IL-6-deficient mice [21]. The authors showed that IL6 knockout mice showed lower BLM-induced inflammatory cell accumulation and reduced fibrotic changes in the lung. Interestingly, the loss of IL6 significantly attenuated the BLM-induced up-regulation of TGF-β1 and chemokine ligand 3 (CCL3). This study suggested that specific drugs targeting IL-6 could be considered potentially effective treatments against PF. More recently, Kobayashi and collaborators have demonstrated that the role of IL-6 signaling could differ between the inflammatory and fibrotic stages of BLM-induced lung injury [22]. Whereas IL-6-neutralizing antibodies ameliorate lung fibrosis at early fibrotic stages of BLM-induced lung injury, blockade of the IL-6 pathway accelerates the development of lung fibrosis at the early inflammatory stage in the BLM-induced PF mice model [22]. This study highlighted the high complexity of the IL-6 signaling by identifying for the first time the dual anti/pro-fibrosis role of IL-6 in the pathogenesis of lung fibrosis. Indeed, further studies have demonstrated that IL-6 potentiates TGF-β signalling by promoting Small Mothers Against Decapentaplegic (SMAD) 3 activation in a STAT3-dependent manner, which increases collagen expression [23].

Calcium homeostasis is a fundamental component in the regulation of gene expression in human pulmonary fibroblasts [24], and its dysregulation plays a central role in fibroblast-mediated myofibroblast differentiation and synthetic/secretory function [25]. Previous studies have demonstrated that pharmacological disruption of calcium signaling improves lung function in the bleomycin-induced IPF model in mice by preventing extracellular matrix deposition, soluble collagen, and hydroxyproline content, an indicator of collagen deposition [26]. The sarcoplasmic reticulum Ca^2+^-ATPase 2a (SERCA2a) plays a major role in calcium homeostasis [27]. Under basal conditions, SERCA2a sequesters intracellular calcium in the sarco/endoplasmic reticulum. The down-regulation of SERCA2a increases intracellular calcium [Ca^2+^]i levels and plays a critical role in heart failure, vascular remodeling diseases, and lung diseases such as pulmonary hypertension [27–31]. Given the essential role of SERCA2a in calcium homeostasis and the beneficial effect of SERCA2a gene transfer in lung diseases, we sought to investigate the role of SERCA2a in PF and decipher the underlying molecular pathway.

**Figure 2:**
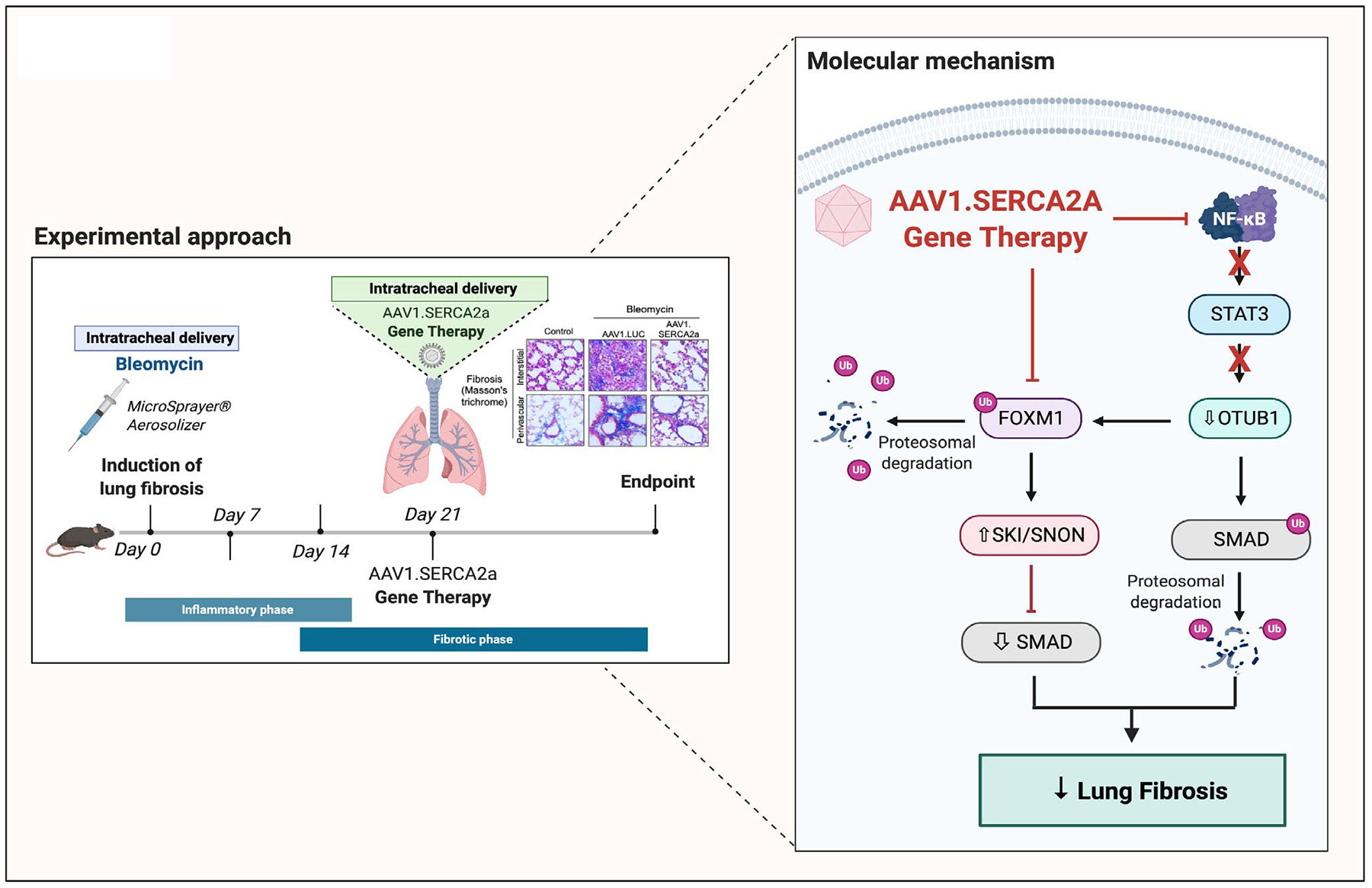
Schematic representation of the experimental approach and molecular mechanisms by which SERCA2a inhibits lung fibrosis. Adapted from Bisserier et al. [32]. Using the bleomycin-induced IPF model, our group previously showed that restoration of SERCA2a expression in lungs by AAV1-mediated gene therapy via intra-tracheal delivery inhibits pulmonary fibrosis *in vivo*. Our study showed that SERCA2a overexpression attenuated the NF-κB/STAT3 activation, which subsequently inhibited the OTUB1/FOXM1 axis. Loss of OTUB1 expression counteracted the SMAD signaling and promoted the expression of the anti-fibrotic SNON and SKI proteins. Altogether, our results showed that lung targeted-SERCA2a gene therapy inhibits lung fibrosis in experimental model of pulmonary fibrosis in rodents. Created with BioRender.com.

In our recent study published in *Molecular Therapy*, our group has investigated the role of SERCA2a gene therapy in lung fibrosis [32]. We found that SERCA2a was drastically decreased in lung biopsies from patients with IPF and in the mouse model of PF induced by bleomycin (Figure 2). Our results also revealed that local intratracheal delivery of AAV1.SERCA2a significantly inhibited lung fibrosis and improved lung function in the BLM-induced PF mouse model [32]. AAV1. SERCA2a effectively reversed pulmonary interstitial and perivascular fibrosis as well as pulmonary vascular remodeling.

At the cellular and molecular level, we demonstrated that SERCA2a inhibited the proliferation, migration, and differentiation of fibroblasts to myofibroblasts *in vitro* by blocking the OTUB1/Forkhead Box M1 (FOXM1) axis and restoring the SNON/SKI pathway, and therefore SMAD2/3 activity (Figure 2) [32]. The SKI (Sloan-Kettering Institute) and SNON (Ski novel) proto-oncoproteins are described as SMAD-interacting proteins that inhibit the TGF-β signalling pathway by disrupting the formation of R-Smad/Smad4 complexes, as well as by impairing SMAD association with the p300/CBP coactivators [33]. It has been previously shown that SNON and Ski are rapidly-degraded in response to TGF-β stimulation [34]. Hyper-activation of the TGF-β pathway, with a decrease of Ski and SNON protein expression due to proteosomal degradation, has been shown in the kidney fibrosis model induced by unilateral ureteral obstruction [35]. *In vitro*, Ski and SnoN overexpression inhibits the profibrotic activity while SnoN inhibition restores the sensitivity to TGF-β signaling in proximal tubular kidney epithelial cells (HKC-8) [36,37]. Consistent with our previous publication, Herhaus et al. have demonstrated that the deubiquitinase OTU domain, ubiquitin aldehyde binding 1 (OTUB1), enhances TGFβ signalling by inhibiting the ubiquitination and degradation of active SMAD2/3 [38]. In 2020, another study demonstrated that OTUB1 potentiated NF-κB-dependent immune responses in dendritic cells by stabilizing the E2-conjugating enzyme UBC13 [39].

Another interesting finding from our group is that SERCA2a blocks NFκB-mediated IL-6 expression *in vitro* in human lung fibroblasts [32]. Therefore, the restoration of SERCA2a may also inhibit inflammatory responses during the earlier phase of lung fibrosis by blocking the OTUB1 and NF-κB-dependent immune signaling. Furthermore, we have uncovered a new molecular mechanism mediated by the transcription factor FOXM1 in lung fibroblasts (Figure 2). FOXM1 is a central component of the nuclear retention of the SMAD3/SMAD4 complex in TGF-β signaling in metastasis [40]. SERCA2a-mediated OTUB1 inhibition promotes the ubiquitination and degradation of FOXM1 (Figure 2). Consistent with this study, Perke et al. found higher FOXM1 mRNA and protein levels in IPF fibroblasts isolated from patients and BLM-induced PF [41]. By upregulating the pro-inflammatory cytokines CCL2, CXCL5, and IL-1β, FOXM1 promotes lung inflammation and proliferation of myofibroblasts [41]. In this study, the authors demonstrated that FOXM1 plays a key role in lung fibroblast activation and fibrogenesis. Using a translational approach, the authors showed that genetic deletion of FOXM1 in fibroblasts or pharmacological inhibition of FOXM1 inhibitor with Siomycin A attenuates BLM-induced pulmonary fibrosis [41]. Thus, inhibition of the OTUB1/FOXM1 by SERCA2a may have a dual beneficial effect by suppressing both the inflammation and fibrotic signaling pathways in IPF.

Finally, *in vivo* results show that lung-targeted AAV1. SERCA2a therapy may be a promising approach for the prevention and treatment of BLM-induced lung damage and/or interstitial PF (Figure 2). Our group demonstrated that intratracheal delivery of aerosolized AAV1 carrying the human SERCA2a gene (AAV1.SERCA2a) decreased lung fibrosis and vascular remodeling after lung injuries in an experimental mouse model of PF induced by BLM [32]. Collectively, this work suggests for the first time that SERCA2A may be a novel and druggable target in IPF and demonstrated that aerosolized gene therapy via intratracheal delivery might be an effective tool in lung disease.

Considerable progress has been made over the past decades in the field of gene therapy to optimize the cell-specificity, lower the immunogenicity, and identify new delivery methods and technology to increase the transduction efficiently. Adeno-associated virus (AAV)-based gene transfer has shown encouraging results with long-term transduction in rodents and large animals with low immunogenicity, with no integration into the host genome and a strong ability to infect dividing/non-dividing cells in various tissues. Recent advances in designing and engineering AAV vectors have contributed to the enhancement of *in vivo* tissue-tropisms of the AAV serotype vectors and therefore reduce the off-target effect while improving the transduction efficiency.

Initially, studies have evaluated the therapeutic potential of SERCA2a gene transfer in the ventricular myocardium and shown promising results in congestive heart failure clinical trials [42,43]. Interestingly, our group has also shown that SERCA2a restoration using AAV1-based gene therapy prevented and reversed the development of pulmonary hypertension in small and large animal models [29–31]. First, we found a significant decrease in SERCA2a protein levels in human lung homogenate samples from PAH patients. We demonstrated that SERCA2a overexpression inhibits Human Pulmonary Artery Endothelial (hPAEC) and smooth muscle cells (hPASMC) proliferation by restoring eNOS activation and inhibiting the NFAT/STAT3 pathway [29]. Furthermore, several other studies have also demonstrated that SERCA2a gene transfer via intratracheal delivery of aerosolized AAV1 carrying the human SERCA2a gene (AAV1.SERCA2a) inhibits PAH in the MCT-induced PAH rat model and chronic post-capillary pulmonary hypertension in a large animal model [29,30]. In the MCT-induced PAH, intratracheal delivery of AAV1.SERCA2a decreased the right ventricular systolic pressure (RVSP), pulmonary artery pressure (PAP), vascular remodeling, right ventricular hypertrophy (Fulton Index), and RV fibrosis in comparison with MCT-PAH rats treated with a control AAV1.β-galactosidase or saline solution [29]. In the prevention protocol, AAV1.SERCA2a delivery successfully attenuated adverse hemodynamic profiles as well as indices of pulmonary and cardiac remodeling in comparison with rats treated with AAV1.β-galactosidase or a saline solution [29].

More recently, safety and long-term efficacy of AAV1. SERCA2a delivery using a nebulizer has been examined in a Yukatan miniature swine model of chronic pulmonary hypertension [44]. Similar to Yorkshire pigs, Yukatan miniature swine developed PH two months after the pulmonary vein banding surgery, as demonstrated by elevated pulmonary pressures, increased vascular resistance, and RV failure [44]. The authors assessed the therapeutic efficacy of nebulized AAV1.SERCA2a at two months after delivery and found that nebulized AAV1.SERCA2a gene therapy significantly decreased pulmonary vascular medial thickness, pulmonary vascular resistance, and increased long-term survival compared to control animals [44].

In summary, our study identified lung-targeted SERCA2a gene therapy as a promising strategy for treating patients with lung fibrosis (Figure 2). Although we have only characterized the anti-fibrotic effects of SERCA2a gene transfer in a BLM-induced PF model, part of the beneficial effects of SERCA2a in PF may be due to its anti-inflammatory properties. Further investigation should be considered to fully evaluate the immunosuppressive properties of SERCA2a in inflammatory lung diseases, including asthma, chronic obstructive pulmonary disease (COPD) and pneumonia. In conclusion, we believe that aerosolized AAV1-based gene therapy via intratracheal delivery represents an attractive therapeutic approach in lung diseases and may be a promising tool for genetic-related respiratory diseases.
